# Reliability of assessing proximal femur geometry with Rutz classification schema in patients with cerebral palsy

**DOI:** 10.1097/BPB.0000000000001077

**Published:** 2022-03-13

**Authors:** Maciej Kasprzyk, Aleksander Koch, Lukasz M. Karbowski, Marek Jóźwiak, Unni G. Narayanan

**Affiliations:** a Department of Pediatric Orthopaedics and Traumatology, Poznan University of Medical Sciences, Poznan, Poland; b Division of Orthopaedic Surgery, The Hospital for Sick Children, University of Toronto, Toronto, Canada

**Keywords:** cerebral palsy, femoral head, hip dislocation, muscle spasticity, neuromuscular diseases, X-rays

## Abstract

Our investigation aimed to assess the reliability of the femoral head shape classification system devised by Rutz *et al*. and observe its application in patients with cerebral palsy (CP) at different skeletal maturity levels. Four independent observers assessed anteroposterior radiographs of the hips of 60 patients with hip dysplasia associated with non-ambulatory CP (Gross Motor Function Classification System levels IV and V) and recorded the femoral head shape radiological grading system as described by Rutz *et al*. Radiographs were obtained from 20 patients in each of three age groups: under 8 years, between 8 and 12 years and above 12 years old, respectively. Inter-observer reliability was assessed by comparing the measurements of four different observers. To determine the intra-observer reliability, radiographs were reassessed after a 4-week interval. Accuracy was checked by comparing these measurements with the assessment of expert consensus. Validity was checked indirectly by observing the relationship between the Rutz grade and the migration percentage. The Rutz classification system’s evaluation of femoral head shape showed moderate to substantial intra- and inter-observer reliability (mean *κ* = 0.64 for intraobserver and mean *κ* = 0.5 for interobserver). Specialist assessors had slightly higher intra-observer reliability than trainee assessors. The grade of femoral head shape was significantly associated with increasing migration percentage. Rutz’s classification was shown to be reliable. Once the clinical utility of this classification can be established, it has the potential for broad application for prognostication and surgical decision-making and as an essential radiographic variable in studies involving the outcomes of hip displacement in CP. Level of evidence: III.

## Introduction

Hip displacement is one of the most commonly observed consequences of muscle imbalance and abnormal bony morphology of the proximal femur in patients with bilateral cerebral palsy (CP). Its incidence ranges from 1 to 75% and is related to the functional level of the patient assessed by the Gross Motor Function Classification System (GMFCS) [1,2]. Untreated progressive hip displacement negatively impacts a child’s health related quality of life . It may contribute to contractures, an inefficient gait in ambulatory children, cause pain, and difficulty in caregiving, including dressing and perineal hygiene in non-ambulatory children [3].

**Fig. 1:**
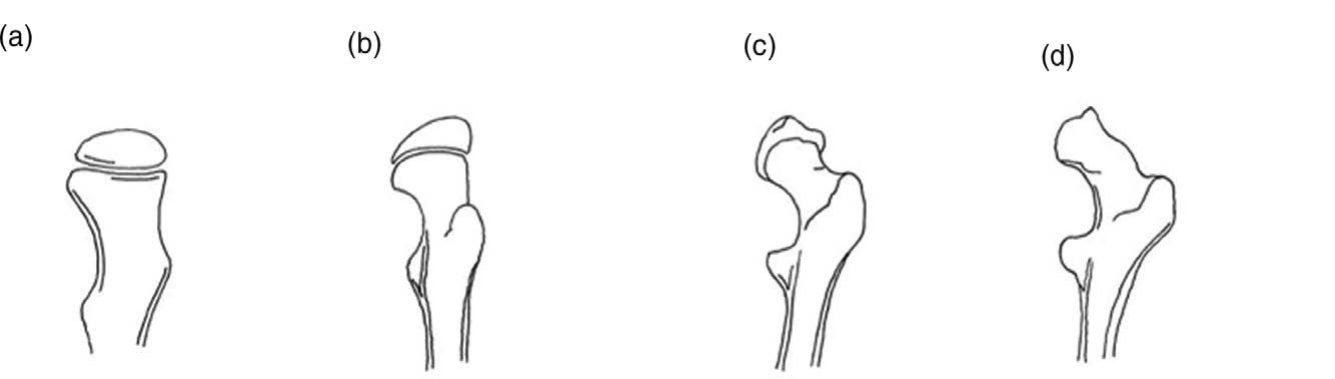
Femoral head shape classification described by Rutz *et al*. The head gradually flattens with the loss of the typical round shape.

**Fig. 2:**
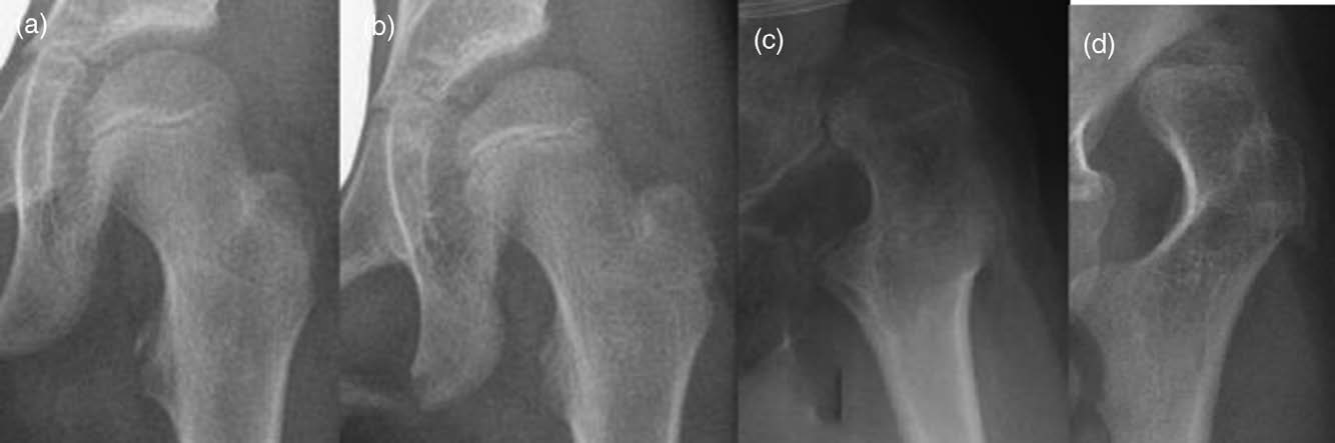
Femoral head shape classification described by Rutz *et al*. demonstrated on AP X-rays. AP, anteroposterior.

The term ‘spastic hip disease’ encompasses all pathological changes within the hip joint due to disturbed muscle balance due to spasticity and abnormal bony morphology; however, hip displacement may occur in this population even in the absence of spasticity and with other movement disorders. The development of acetabular deficiency or dysplasia arises from abnormal forces on the acetabulum associated with increasing lateral, superior and posterior displacement of the femoral head. The femoral head gradually migrates outside the acetabulum [4]. Clinical evaluation of the hip is not a reliable indicator of hip displacement, so hip morphology in CP should be routinely assessed and monitored using anteroposterior (AP) radiographs of the pelvis. The literature describes several scales to assess the geometry of the proximal femur [5,6]. Recently, Rutz and colleagues [7] presented their own femoral head shape grading system for patients with CP. This classification is a revised and simplified version of the better known classification Melbourne Cerebral Palsy Hip Classification System (MCPHCS) [5]. Type A is a typically spherical femoral head with intact cartilage. Type B has medial flattening and a head-in-neck deformity. Type C is a progression from type B in which a lateral trough is also present. Type D is consistent with the complete loss of the round shape. (Figs. 1 and 2).

Our study aims to assess the reliability of the Rutz classification system of femoral head shape in patients with CP of different ages up to skeletal maturity using AP radiographs of the hips.

## Materials and methods

### Study population

The study was conducted with the approval of the Institutional Review Board. Four independent observers (two consultants and two residents) assessed both hips on 60 pelves AP X-rays of 60 non-ambulatory (GMFCS IV, V) patients (31 male, 29 female) with known ‘spastic’ hip disease associated with CP from the authors’ institution, who had been admitted to the hospital for operative management. Patients with neuromuscular diseases other than CP were excluded. An AP pelvis radiograph was made with the patient in the supine position with the hips in neutral abduction/adduction and rotation and the legs held parallel to each other.

### Clinical features

Patients were all non-ambulatory and included both GMFCS levels IV and V. All X-rays were assessed with the femoral head shape radiological grading system described by Rutz *et al* [7]. Researchers are fluent in English and read original articles on the Rutz and MCPHCS classification. An equal number of patients were included from three age groups: under 8, between 8 and 12 years old and above 12 years old, respectively. Radiographs were reassessed after a 4-week interval by all four observers to determine the intra-observer reproducibility. For the second assessment, the radiographs were presented in random order. Each observer was blinded to the assessments performed by the other observers and to their first assessments. Reimer’s migration percentage of each hip was also obtained and evaluated by selected observers. If the lateral margin of the acetabulum deforms and looks like a ‘gothic arch’ as in the advanced stages of the disease, we used the midpoint (apex) of the Gothic arch to mark the lateral acetabular margin instead of Perkin’s line [3,8,9]. Hip subluxation was defined as a migration percentage greater than 30% and lesser than 100%, with hip dislocation defined as a migration percentage equal to 100%. We also assessed whether dislocated hips overlapped with the iliac bone. Observation of this overlap affecting the evaluation was also considered. Patients’ demographics and clinical characteristics are presented in Table 1.

**Table 1:** Patient demographics and clinical characteristics with cerebral palsy

Patient demographics				
	Overall (*n* = 60)	Group I (*n*1 = 20)	Group II (*n*2 = 20)	Group III (*n*3 = 20)
Mean age (SD) and range	10 years, 3 months (SD: 3 years, 1 month); Range 5 years–16 years, 1 month	7 years, 1 month (SD: 1 year, 5 months); Range 5 years–7 years, 8 months	9 years, 10 months (SD: 1 year, 2 months); Range 8 years 6 months–11 years, 2 months	13 years, 11 months (SD: 1 year, 6 months); Range 12 years 4 months–16 years, 1 month
Sex	31M, 29F	8M, 12F	11M, 9F	12M, 8F
GMFCS IV	30 (50%)	12 (60%)	7 (35%)	11 (55%)
GMFCS V	30 (50%)	8 (40%)	13 (65%)	9 (45%)
Femoral head overlapped by pelvis	7R, 5L	0R, 0L	4R, 3L	3R, 2L
Migration percentage R, L: mean (SD)	58% R, 60% L (SD: 36% R, 24% L)	58% R, 74% L (SD: 32% R, 31% L)	61% R, 62% L (SD: 32% R, 39% L)	55% R, 39% L (SD: 38% R, 35% L)
Hip dislocation migration percentage = 100	20R, 24L	6R, 11L	7R, 9L	7R, 4L
Hip subluxation migration percentage > 30 < 100	26R, 23 L	11R, 8 L	9R, 6 L	6R, 9 L
Stable hip joint migration percentage < 30	14R, 12L	3R, 1L	4R, 5L	7R, 7L

GMFCS, Gross Motor Function Classification System.

### Reliability measures

The inter-rater and intra-rater reliability of the ordinal variable of the grading system was determined with the weighted Cohen kappa statistic and 95% confidence interval to estimate the error to be expected in single measurements. The influence of age, sex, GMFCS level, observer experience (specialist vs. resident), and migration percentage on the reliability was also analysed.

### Validity

In order to validate the Rutz classification, we examined the relationship between the classification grade and the migration percentage. Because increasing migration percentage has been associated with worse outcomes, this relationship indirectly validates the Rutz classification’s clinical significance. We hypothesized that by increasing the grade of the Rutz classification, the migration percentage would be greater. This was tested by comparing the mean migration percentage in the four grades of the classification system, using one Kruskal–Wallis test with the Dunn–Bonferroni test of multiple comparisons and Jonckheere–Terpstra test for the trend (to assess whether the value of expert judgment increases with increasing migration value). In the case of the inter-observer agreement, we computed the Mann–Whitney test, Spearman’s rank correlation coefficient (for demographic variables), and Cohen’s kappa coefficient (linear weights). Statistica 13 (TIBCO Software Inc., Palo Alto, California, USA) and PQStat (PQStat Software, Poznań/Plewiska, Poland) were used.

## Results

The patients had a mean (SD) age of 10 years, 3 months (SD: 3 years, 1 month). There were 29 girls and 31 boys. The average (SD) migration percentage for the right and left hips were 58% and 48% (SD: 36, 24%), respectively, which included hips with normal migration percentage to complete dislocation (migration percentage = 100%). The GMFCS level distribution is noted in Table 1. We included the distribution of femoral head deformity among the observed groups in Table 2.

**Table 2:** Distribution of femoral head deformity among the observed groups

Femoral head according to Rutz classification	Group I (age range 5 years–7 years, 8 months)	Group II (age range 8 years 6 months–11 years, 2 months)	Group III (age range 12 years 4 months–16 years, 1 month)
A	12 R 12L	8R 13L	9R 10L
B	8R 8L	11R 7L	8R 7L
C	0R 0L	1R 0L	3R 2L
D	0R 0L	0R 0L	0R 1L

The Rutz classification of the femoral head shape classification system showed moderate to substantial intra-observer reliability and moderate inter-observer reliability. The mean intra-observer reliability overall for specialists and trainees was 0.64 (substantial), while the mean inter-observer reliability was 0.5 (moderate). Specialists demonstrated higher intra-observer reliability than resident assessors, with both groups having similar levels in kappa cohen statistics for both hip joints. (Table 3) The right hip joint evaluation showed higher intra-observer reliability than the left hip, but this observation was not statistically significant.

**Table 3:** Intra-observer and inter-observer reliability of assessment by the Rutz classification

Reliability	Weighted Cohen kappa for right joint (95% CI)	Weighted Cohen kappa for left joint (95% CI)
Intraobserver		
First consultant	0.74 (0.569–0.903)	0.66 (0.498–0.815)
Second consultant	0.69 (0.520–0.868)	0.66 (0.480–0.840)
First resident	0.70 (0.529–0.871)	0.63 (0.437–0.830)
Second resident	0.63 (0.458–0.807)	0.67 (0.516–0.815)
Interobserver		
Specialist group	0.49 (0.283–0.688)	0.50 (0.308–0.689)
Resident group	0.63 (0.471–0.780)	0.41 (0.240–0.569)

CI, confidence interval.

Neither the GMFCS level nor the sex of the patient influenced the intra- or inter-observer measurement reliability in both specialist and resident groups. migration percentage (subluxation or dislocation) did not influence the reliability of the assessments; however, the intra-rater reliability of assessments was higher in older patients. The overlapping of the ilium over the femoral head reduced the inter-rater reliability in both specialist and resident groups and significantly more so in the resident assessments (Table 4).

**Table 4:** Inter-observer reliability of assessment by the Rutz classification with and without overlapping of the ilium over the femoral head

	Weighted Cohen kappa for right joint with overlapping (95% CI)	Weighted Cohen kappa for right joint without overlapping (95% CI)	Weighted Cohen kappa for left joint with overlapping (95% CI)	Weighted Cohen kappa for left joint without overlapping (95% CI)
Specialist group	0.46 (0.24–0.68) *P* < 0.01	0.53 (0.144–0.922) *P* < 0.05	0.477 (0.27–0.684) *P* < 0.01	0.705 (0.305–1.105) *P* < 0.05
Resident group	0.39 (−0.129–0.912) *P* > 0.14	0.65 (0.506–0.795) *P* < 0.01	0.397 (0.207–0.586) *P* < 0.01	0,444 (0.179–0.709) *P* < 0.05

CI, confidence interval.

Increasing the grade of the Rutz classification for all observers was found to be statistically significantly associated with increasing migration percentage, as hypothesized, providing some evidence of the validity of the classification. (*P* < 0.01 in the Jonckheere–Terpstra test for ordered alternatives).

## Discussion

Although the primary neurologic deficit in CP is permanent and nonprogressive, the musculoskeletal consequences, including those to the lower extremity, are progressive over the growing years. The untreated hip’s natural pattern of progressive subluxation is usually associated with skeletal deformation of the proximal femur. Because clinical examination of the hip cannot provide reliable information about hip displacement or femoral head shape [3,10], radiographic evaluation of the hips is necessary for patients with CP who are at high risk for hip displacement. The spastic adductors and hip flexors produce forces that result in the distortion of the femoral head against the posterolateral acetabulum and labrum: the acetabulum capsule and superior rim of the acetabulum can cause focal deformation of the femoral head. The uncovered portion of the femoral head is also subjected to the forces of the overlying muscles, including the rectus femoris, gluteus medius and minimus [11,12]. Moreover, there are differences in the forces acting in ambulatory and non-ambulatory patients. The indented femoral head abuts on the acetabular edge, with the progression of cartilage loss. These changes are usually associated with pain over time. Reliable quantification of the shape of the femoral head is, therefore, of great value. Previous studies have suggested establishing a concentric femoral head within the acetabulum before age 5 for proper hip joint development [10,13]. On the other hand, it is plausible that some loss of shape might still be reversible following appropriate hip reconstructive surgery due to remodelling, while more severe deformities of the femoral head with permanent loss of cartilage might be candidates for salvage surgery if associated with severe pain. Rutz *et al*. [14] showed that the femoral head could gradually remodel after hip reconstruction surgery in non-ambulatory patients. That is observed by using the Rutz classification rather than the Mose technique. This points out that resection of the femoral head may not be indicated because of the tendency for the hip joint to become more congruent and spherical.

For a classification system to be helpful, it must first be shown to be reliable or reproducible. Our study has demonstrated that the Rutz classification of the femoral head shape showed moderate to substantial reliability. The results obtained are lower than in the more sophisticated MCPHCS classification. A standardized radiographic technique must be followed to ensure reliability between interval radiographs and patients [15]. Inappropriately performed AP radiographs can result in a less reliable evaluation of the hip [15].

Reliability alone is insufficient. The classification system must also be shown to be accurate and valid. In our study, we found, as hypothesized, that worsening grade of femoral head shape should be associated with increasing displacement as measured by the migration percentage. migration percentage has been proven to be prognostically helpful and is the crucial factor influencing surgical decision-making and measuring radiographic outcomes. Adding the femoral head shape provides additional detail that might influence prognosis and decision-making.

Before discussing the clinical implications of the present study, it is essential to address the study’s limitations. First, there might be a debate regarding the femoral head geometry assessment based on two-dimensional radiographs compared to computed tomography (CT) scans; however, the Rutz classification is based on AP radiographs, not CT scans. Hip surveillance programs [16,17] recommend standardized AP radiographs of the pelvis with the child in a supine position. AP radiographs are easier to obtain, more readily available, less costly and associated with lower doses of radiation for patients. CT scans are not as universally or readily available, require sedation in some children, and the dose of radiation for contrasts is 10–20 times higher than from a single AP radiograph of the pelvis. Gose *et al*. [18] found excellent concurrent validity between migration percentage and the degree of migration in the frontal plane using a three-dimensional migration model based on three-dimensional CT.

The target population within our study included a diverse spectrum of patients with CP with a wide range of ages; both sexes represented and both non-ambulatory GMFCS levels with a full range of hip migration percentages. This allowed us to evaluate the effects of all these factors, including the GMFCS level, the magnitude of hip displacement (migration percentage), sex and age of patients and the overlapping of the hip joint by the pelvis, on the reliability of the classification system. Because most patients had some hip displacement, care should be taken when applying these results to patients with normal hip joints.

There are many radiographic scales in the literature for assessing surgical treatment results of hip interventions, most of which have been applied in developmental hip dysplasia. Robin *et al*. [5] developed a new classification system of hip disease in CP (MCPHCS), showing excellent inter- and intra-observer reliability [19]. Gose *et al*. evaluated the validity of the MCPHCS in younger individuals with CP using three-dimensional CT to compare with other populations; however, the MCPHCS was designed for skeletally mature individuals with CP and was intended to assess radiographic outcomes based on the developed deformities or postoperative distorted features. This approach includes the assessment of Reimer’s Migration Index, continuity of Shenton’s arch, evaluation of roundness of the femoral head and assessment of the acetabulum development and pelvic obliquity. Although it shows excellent intra- and inter-rater reliability, the large number of parameters makes assessing more difficult. The Rutz classification of the femoral head shape is a more straightforward, intuitive classification independent of the migration percentage and the classification of the morphology of the proximal femur. Both classification schemas have clinical prognostic value; however, Robin’s classification was primarily intended for hips at skeletal maturity and as a radiographic outcome rather than for decision-making that usually occurs long before skeletal maturity. Studies that adapted this classification for children between 2 and 7 years of age lacked classes I and IV in the grading system. As a result, Rutz’s classification is more universally applicable. When considering Rutz’s classification, the agreement between ‘estimated’ hip grade and good hip grade based on migration percentage was good to excellent. The rate of agreement between the four qualitative morphological features of the classification and the hip grading based on migration percentage was reflective of observed deformations [20]. Although we have included information on the possible use of the classification in skeletally immature patients, the obtained results do not fully meet the expectations. Ulusaloglu *et al*. [21] showed an increase in the number of type C and D femoral head shapes in older children with CP close to skeletal maturity. Observed changes can even progress after skeletal maturity. They also found migration percentage higher than ~30% at triradiate cartilage closure as the cut-off point over which the possibility of severe deformity increases.

Pons’ systematic review on the validity and reliability of different radiological methods in assessing proximal femur geometry in children with CP showed significant variability between current assessments [22]. The migration percentage on AP hip radiographs showed excellent reliability and concurrent validity with CT scan measurements. The author also assessed the acetabular index and neck-shaft angle, showing reliability and validity. The validity and reliability of femoral anteversion were assessed by Chung [23], but Pons *et al*. suggest the necessity for more studies on this subject matter.

Although our study has shown acceptable reliability of the Rutz classification, its validation will require evaluation of its clinical utility. In Rutz’s [7] study, the preoperative migration percentage was the most influential risk factor affecting the postoperative outcome, showing an association with pain intensity and suggesting performing hip reconstruction early, before a migration percentage increase is observed. In our study, increasing the classification grade was associated with increasing migration percentage, which indirectly validated the classification system; however, true validation would require an exploration of the classification grade with the presence of hip symptoms or health-related quality of life to truly understand its clinical value. This would require testing how well the classification is related to the prognosis of hip health, such as pain/comfort, flexibility/stiffness, and ease of care in non-ambulatory patients or pain and mobility in ambulatory patients. Moreover, even authors of classification [14] indicate a need for previous assessment of reliability and validity as one of the limitations of their system.

The Rutz study suggested that preoperative head shape had no significant effect on pain intensity and frequency changes [7]. Current literature prefers head-preserving treatment over resection arthroplasty [24], which provides some evidence for the reconstruction of a hip, even with a deformed femoral head, to alleviate pain; however, there is likely to be some upper limit of deformity that precludes an effective reconstructive strategy. The ongoing CP Hip Outcomes Project is a large international multicentre cohort study that will be testing prospectively the association between the grade of femoral head shape and the presence of hip-related symptoms or health-related quality of life as measured by the Caregiver Priorities and Child Health Index of Life with Disabilities [25], a validated measure of health related quality of life for this population; as well as the additional impact of femoral head shape (beyond migration percentage alone) on the outcomes of reconstructive versus salvage strategies.

If shown to have this clinical utility, this classification system has the potential for many future applications: for instance, to better quantify the natural history of untreated hip displacement, to study the influence of intervention to preserve or alter the shape of the femoral head favourably in growing children with CP, and guide the better selection of patients who would or would not benefit from hip reconstruction. Additionally, the classification schema may show potential in enhancing diagnostic properties and analyzing the remodelling potential of the femoral head post-surgical treatment of patients with CP.

In summary, our study has demonstrated that the Rutz classification of the femoral head shape has moderate to substantial intra-observer reliability and moderate inter-observer reliability. This classification system might be helpful to incorporate or report within routine radiographic hip surveillance in CP, mainly if its value for prognostication and to guide surgical decision-making can be established.

## Acknowledgements

We would like to acknowledge the contribution of the CP Hip Outcomes Project Study Group.

### Conflicts of interest

There are no conflicts of interest.

## References

[1] Soo B Howard JJ Boyd RN Reid SM Lanigan A Wolfe R. Hip displacement in cerebral palsy. J Bone Joint Surg Am 2006; 88:121–129. 16391257 10.2106/JBJS.E.00071

[2] Palisano R Rosenbaum P Walter S Russell D Wood E Galuppi B. Development and reliability of a system to classify gross motor function in children with cerebral palsy. Dev Med Child Neurol 1997; 39:214–223. 9183258 10.1111/j.1469-8749.1997.tb07414.x

[3] Lins LAB Watkins CJ Shore BJ. Natural history of spastic hip disease. J Pediatr Orthop 2019; 39:S33–S37. 31169645 10.1097/BPO.0000000000001347

[4] Morrell DS Pearson JM Sauser DD. Progressive bone and joint abnormalities of the spine and lower extremities in cerebral palsy. Radiographics 2002; 22:257–268. 11896216 10.1148/radiographics.22.2.g02mr19257

[5] Robin J Kerr Graham H Selber P Dobson F Smith K Bakeret R. Proximal femoral geometry in cerebral palsy: a population-based cross-sectional study. J Bone Jt Surg Br 2008; 90:1372–1379. 10.1302/0301-620X.90B10.20733 18827250

[6] Miller F Girardi H Lipton G Ponzio R Klaumann M Dabney KW. Reconstruction of the dysplastic spastic hip with peri-ilial pelvic and femoral osteotomy followed by immediate mobilization. J Pediatr Orthop 1997; 17:592–602. 9591996 10.1097/00004694-199709000-00005

[7] Rutz E Vavken P Camathias C Haase C Jünemann S Brunner R. Long-term results and outcome predictors in one-stage hip reconstruction in children with cerebral palsy. J Bone Jt Surg Am 2015; 97:500–506. 10.2106/JBJS.N.00676 25788307

[8] Kim SM Sim EG Lim SG Park ES. Reliability of hip migration index in children with cerebral palsy: the classic and modified methods. Ann Rehabil Med 2012; 36:33–38. 22506233 10.5535/arm.2012.36.1.33 PMC3309325

[9] Shore B Spence D Graham HK. The role for hip surveillance in children with cerebral palsy. Curr Rev Musculoskelet Med 2012; 5:126–134. 22430862 10.1007/s12178-012-9120-4 PMC3535157

[10] Kalen V Bleck EE. Prevention of spastic paralytic dislocation of the hip. Dev Med Child Neurol 1985; 27:17–24. 3979669 10.1111/j.1469-8749.1985.tb04520.x

[11] Abel MF Wenger DR Mubarak SJ Sutherland DH. Quantitative analysis of hip dysplasia in cerebral palsy: a study of radiographs and 3-D reformatted images. J Pediatr Orthop 1994; 14:283–289. 8006155 10.1097/01241398-199405000-00002

[12] Beck M Woo A Leunig M Ganz R. Gluteus minimus induced femoral head deformation in dysplasia of the hip. Acta Orthop Scand 2001; 72:131–117. 10.1080/000164701753606626 11327407

[13] Scrutton D. The early management of hips in cerebral palsy. Dev Med Child Neurol 1989; 31:108–116. 2646162 10.1111/j.1469-8749.1989.tb08419.x

[14] Ma N Tischhauser P Camathias C Brunner R Rutz E. Long-term evolution of the hip and proximal femur after hip reconstruction in non-ambulatory children with cerebral palsy: a retrospective radiographic review. Children (Basel) 2022; 9:164. 35204886 10.3390/children9020164 PMC8869786

[15] Lim S-J Park Y-S. Plain radiography of the hip: a review of radiographic techniques and image features. Hip Pelvis 2015; 27:125–134. 27536615 10.5371/hp.2015.27.3.125 PMC4972716

[16] Shrader MW Wimberly L Thompson R. Hip surveillance in children with cerebral palsy. J Am Acad Orthop Surg 2019; 27:760–768. 30998565 10.5435/JAAOS-D-18-00184

[17] Hägglund G Alriksson-Schmidt A Lauge-Pedersen H Rodby-Bousquet E Wagner P Westbom L. Prevention of dislocation of the hip in children with cerebral palsy: 20-year results of a population-based prevention programme. Bone Joint J 2014; 96B:1546–1552. 10.1302/0301-620X.96B11.34385 25371472

[18] Gose S Sakai T Shibata T Murase T Yoshikawa H Sugamoto K. Morphometric analysis of the femur in cerebral palsy: 3-dimensional CT study. J Pediatr Orthop 2010; 30:568–574. 20733422 10.1097/BPO.0b013e3181e4f38d

[19] Murnaghan ML Simpson P Robin JG Shore BJ Selber P Graham HK. The cerebral palsy hip classification is reliable: an inter- and intra-observer reliability study. J Bone Joint Surg Br 2010; 92:436–441. 20190318 10.1302/0301-620X.92B3.23105

[20] Robin J Graham HK Baker R Selber P Simpson P Symons S. A classification system for hip disease in cerebral palsy. Dev Med Child Neurol 2009; 51:183–192. 19055594 10.1111/j.1469-8749.2008.03129.x

[21] Ulusaloglu A Asma A Rogers K Shrader MW Miller F Howard JJ. Femoral head deformity associated with hip displacement in nonambulatory cerebral palsy: results at skeletal maturity. J Pediatr Orthop 2023; 43:156–161. 36563091 10.1097/BPO.0000000000002333

[22] Pons C Rémy-Néris O Médée B Brochard S. Validity and reliability of radiological methods to assess proximal hip geometry in children with cerebral palsy: a systematic review. Dev Med Child Neurol 2013; 55:1089–1102. 23731365 10.1111/dmcn.12169

[23] Lee KM Kang JY Chung CY Kwon DG Lee SH Choi IH. Clinical relevance of valgus deformity of proximal femur in cerebral palsy. J Pediatr Orthop 2010; 30:720–725. 20864860 10.1097/BPO.0b013e3181edba2a

[24] Boldingh EJ Bouwhuis CB Van Der Heijden-Maessen HCM Bos CF. Lankhorst GJ palliative hip surgery in severe cerebral palsy: a systematic review. J Pediatr Orthop B 2014; 23:86–92. 24025529 10.1097/BPB.0b013e3283651a5d

[25] Narayanan UG Fehlings D Weir S Knights S Kiran S Campbell K. Initial development and validation of the Caregiver Priorities and Child Health Index of Life with Disabilities (CPCHILD). Dev Med Child Neurol 2006; 48:804–812. 16978459 10.1017/S0012162206001745

